# The indirect health impacts of the COVID-19 pandemic on children and adolescents: A review

**DOI:** 10.1177/13674935211059980

**Published:** 2022-03-10

**Authors:** Tina GA Oostrom, Patricia Cullen, Sanne AE Peters

**Affiliations:** 1 Julius Center for Health Sciences and Primary Care, University Medical Center Utrecht, 8124 Utrecht University, Utrecht, The Netherlands; 2 School of Population Health, 7800 UNSW Sydney, Sydney, NSW, Australia; 3 The George Institute for Global Health, 7800 UNSW Sydney, Sydney, NSW, Australia; 4 Ngarruwan Ngadju: First Peoples Health and Wellbeing Research Centre, University of Wollongong, Sydney, NSW, Australia; 5 The George Institute for Global Health, 4615 Imperial College London, London, UK

**Keywords:** Children’s rights, epidemiology, psychology, vulnerability

## Abstract

It is pertinent to examine potentially detrimental impacts of the coronavirus disease 2019 (COVID-19) pandemic on young people. We conducted a review to assess the health impacts of the COVID-19 pandemic on children and adolescents. Databases of MEDLINE, Embase and the Cochrane Library were searched in June 2020, using keywords for ‘children’, ‘adolescents’ and ‘COVID-19’. English papers discussing young people in context to the COVID-19 pandemic were included. Quality of selected studies was evaluated and scored. Of the 2013 identified articles, 22 met the inclusion criteria, including 11 cohort studies, ten cross-sectional studies and one report. Five main issues emerged: Increased mental health conditions, declines in presentations to paediatric emergency departments, declines in vaccination rates, changes in lifestyle behaviour (mainly decreased physical activity for specific groups of children), and changes in paediatric domestic violence and online child sexual abuse. There are early indications that the COVID-19 pandemic is impacting the health of young people, and this is amplified for those with existing health conditions and vulnerabilities. Despite this, there is limited insight into the protective factors for young people’s health and wellbeing, as well as how the impacts of the pandemic can be mitigated in both the short and long term.

## Introduction

In 2020, a novel coronavirus disease 2019 (COVID-19) caused a pandemic that enforced strict stay-at-home or lockdown orders and social distancing to populations across the world. People are forced to work from home and nurseries, schools, universities are closed. Many sports and social events have been cancelled (Fan et al., 2020; Fegert et al., 2020).

While children are relatively spared from direct health impacts of COVID-19 (Li et al., 2020; Roberton et al., 2020), studies on past health-related crises with similar disease-containment measures show high post-traumatic stress rates among children (Sprang and Silman, 2013). For instance, the Ebola outbreak in West Africa in 2014 had large implications for health care systems, with a 40% reduction of paediatric admissions for malaria and reductions in vaccine coverage (Elston et al., 2017). It also led to higher rates of sexual abuse and neglect of children (Đapić et al., 2020). Likewise, an epidemic involving severe acute respiratory syndrome (SARS) in 2003 resulted in delayed physiological and cognitive development in early childhood (Fan et al., 2020).

Countries and its citizens are facing myriad indirect health effects of COVID-19, including mental health issues (Fegert et al., 2020). However, emphasis is often placed on adults, and children and adolescents are frequently overlooked. Nevertheless, restrictions introduced to manage this pandemic equally enforce major adjustments to children’s daily life, including limitations in key components for normal and healthy development such as social interaction, physical activity and access to school-based education (Saurabh and Ranjan, 2020). Hence, there are growing concerns that the disease-containment measures of COVID-19 may have lasting consequences for future health, wellbeing and development (Brooks et al., 2020; Green, 2020).

Insight into the ramifications for this population has not been provided. However, in order to address and prevent long-term negative outcomes, it is critical to understand the impacts of COVID-19 on young people. Therefore, we undertook a narrative review to assess the impact of the COVID-19 pandemic on child and adolescent health.

### Aim

To identify indirect health impacts of the COVID-19 pandemic on children and adolescents.

## Methods

### Design

A systematic literature search was conducted to identify health impacts of the COVID-19 pandemic on children and adolescents. This review is reported using the Preferred Reporting Items for Systematic Reviews and Meta-Analyses (PRISMA) guidelines for systematic reviews, which were used to inform the protocol for this review.

### Search strategy

The following databases were searched in June 2020: MEDLINE, Embase and the Cochrane Library. COVID-19 has caused a recent and rapid developing pandemic, consequently, preparatory research has shown that there is a scarce amount of evidence covering the entire objective of this review. To collect all available data, our search was focused on article discussing COVID-19 and children/adolescents. Specific subject headings were identified in each database. Search strings combining (adjusted) MeSH Terms, keywords ‘children’, ‘adolescents’ and ‘COVID-19’, and their synonyms were used in MEDLINE and Embase (Supplementary Tables I and II). Additional data were collected by searching reference lists of key sources and generic internet search engines.

### Study selection and inclusion criteria

Titles and abstracts of the selected articles were screened by one author, following predefined inclusion and exclusion criteria (Supplementary Table I). For non-descriptive titles and/or abstracts, full texts were consulted. Finally, full texts of all remaining articles were screened. Many studies focused on the occurrence and characteristics of COVID-19 in children. However, this was not the focus of the review. All other, indirect, health impacts of the pandemic on children and/or adolescents were included.

### Data extraction and analysis

Key characteristics, such as country, study objectives, participant characteristics, outcomes and COVID-19 restrictions were extracted, and indirect health impacts of COVID-19 pandemic on children and adolescents were identified. Using a narrative synthesis approach, the impacts were categorised into themes. Risk of bias analysis was conducted using the Newcastle—Ottawa Quality Assessment Scale for cohort studies (Wells et al., 2006). An adapted version was used for cross-sectional studies (Supplementary Table III) (Wells et al., 2006).

## Results

**Figure 1.**
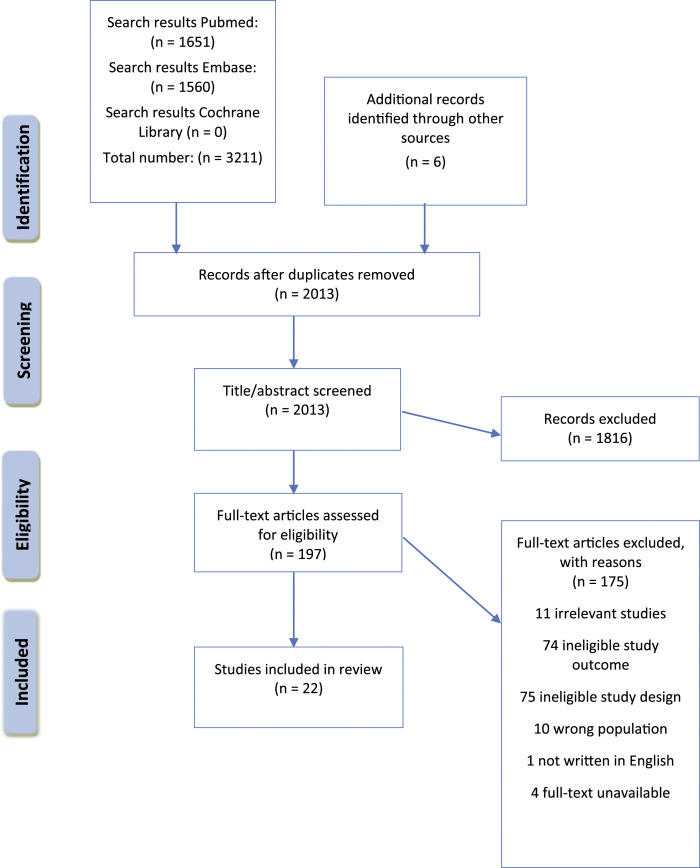
PRISMA flow diagram.

After removing duplicates, 2013 studies were identified, including six found through other sources and searching reference lists. Of these, 197 were retained for full text review and 22 studies were included in the review (Figure 1).

### Study characteristics

Included studies differed in study design, study population and study outcome (Table 1). The age of included children ranged from ≤2 years to 18 years old. Most studies were conducted in China (*n* = 5) (Chen et al., 2020; Li et al., 2020; Xie et al., 2020; Zheng et al., 2020; Zhou et al., 2020), North America (*n* = 5) (Bram et al., 2020; Bramer, 2020; Hemphill et al., 2020; McLay, 2020; Santoli et al., 2020) and Italy (*n* = 4) (Bressan et al., 2020; Buzzi et al., 2020; Colizzi et al., 2020; Pietrobelli et al., 2020). Included studies showed a wide ranged in sample size, from 41 (Pietrobelli et al., 2020) to 871,543 (Angoulvant et al., 2020). The median quality score was 8 (range 3 (Buzzi et al., 2020) to 9 (Bram et al., 2020; Zheng et al., 2020)). Ten studies assessed the mental health outcomes in children and adolescents during COVID-19 (Bobo et al., 2020; Buzzi et al., 2020; Chen et al., 2020; Colizzi et al., 2020; Pınar Senkalfa et al., 2020; Saddik et al., 2020; Saurabh and Ranjan, 2020; Xie et al., 2020; Zheng et al., 2020; Zhou et al., 2020), mostly focused on depression and anxiety rates (Chen et al., 2020; Pınar Senkalfa et al., 2020; Saddik et al., 2020; Xie et al., 2020; Zheng et al., 2020; Zhou et al., 2020). Four studies assessed the impact on vaccination rates (Bramer, 2020; Chandir et al., 2020; McDonald et al., 2020; Santoli et al., 2020) and another four assessed impacts on health care use, including paediatric emergency healthcare department visits (Angoulvant et al., 2020; Bram et al., 2020; Bressan et al., 2020; Li et al., 2020). Two studies reported lifestyle behaviour impacts (Hemphill et al., 2020; Pietrobelli et al., 2020) and another two domestic violence and child abuse (EUROPOL, 2020; McLay, 2020).

**Table 1:** Characteristics of included studies.

Author (year)	Country	Location of study	Study design (timeline)	Sample size, age (mean ± SD), male/female %	Intervention^*^	Outcome(s)	Findings	Risk of bias (NOS)
Impact on mental health								
Bobo et al. (2020)	France	France	Cross-sectional	538 parents (95% female) of children, mean age 10.5 ± 2.93, 86.67%/13.33%	Lockdown (day 20–30 2020)	Wellbeing and global life conditions of children with attention deficit hyperactivity disorder (ADHD)	34.7% worsened, 31.0% improved wellbeing in children with ADHD	7
Buzzi et al. (2020)	Italy	Italy	Cross-sectional (March 2020)	2064 adolescents	Lockdown	Concerns and fears, information on the pandemic and impact on everyday life	Concerns about negative impact on school education in 36.8%	3
Chen et al. (2020)	China	Guiyang, China	Cross-sectional (16–23 April 2020)	1036 adolescents, age 6–15, 531/505	Not specified	Depression and anxiety	18.9% prevalence of anxiety, 11.8% of depression	8
Colizzi et al. (2020)	Italy	Northern Italy	Cross-sectional (6–20 April 2020)	527 parents of children, mean age 13 ± 8.1	Lockdown	Psychosocial and behavioural problems in individuals with autism spectrum disorder	51.5% pre-existing behavioural problems, 41.5% more frequent, 35.5% more intense behavioural problems	8
Saddik et al. (2020)	United Arab Emirates	United Arab Emirates	Cross-sectional (24 March–15 May 2020)	1469 parents and teachers (82.8% female) of children, age 3–16	Not specified	Anxiety	Divorced/separated parents, parents who were teachers and parents with severe anxiety were more likely to report anxiety in their children	8
Saurabh and Ranjan (2020)	India	India	Cross-sectional	121 children, age 9–18 years (+parents), mean age 15.4, 85.12%/30.57%	Home-quarantined and not-quarantined	Assessment of qualitative indicators of psychological impact of quarantine versus no quarantine	Quarantine was associated with several psychological symptoms	6
				131 control children, same age				
Pınar Senkalfa et al. (2020)	Turkey	Turkey	Cross-sectional (15–20 April 2020)	45 children and their mothers, age 0–18, 90 healthy control children and their mothers, same age	Not specified	Anxiety in young people with cystic fibrosis (CF) vs. control group	Lower levels of anxiety among children with CF than non-CF children	7
Xie et al. (2020)	China	Hubei, China	Cross-sectional (28 February–5 March 2020)	1784 students, grade 2–6, 56.7%/43.3%	Lockdown (23 January–8 April 2020 Wuhan, 24 January–23 March 2020 Huangshi)	Depression and anxiety	18.9% prevalence of anxiety, 22.6% of depression	7
Zheng et al. (2020)	China	Hangzhou, China	Cross-sectional	1620 students, age 7–13 (10.10 ± 1.63), 52.2%/47.8%	Lockdown (at least 1 month)	Depression and anxiety	17.2% prevalence of anxiety, 6.3% of depression	9
Zhou et al. (2020)	China	Hubei and 20 other provinces in China	Cross-sectional (8–15 March 2020)	8079 junior and senior high school students, age 12–18 (16), 46.5%/53.5%	Not specified	Depression and anxiety	7.4–37.4% prevalence of anxiety, 10.1–43.7% of depression	8
Impact on domestic violence and child abuse								
EUROPOL (2020)	The Netherlands	Europe	Report	Child sexual abuse material	Lockdown	Numbers of child sexual abuse material distributed, contact made to hotlines	Increased distribution of online child sexual abuse material and amounts of phone calls to hotlines	-
McLay (2020)	UK	Chicago, US	Retrospective cohort (March 2020 vs. March 2019)	4618 reports	Lockdown	Domestic violence during pandemic and during shelter-in-place order	Decreased reported cases of child abuse (*n* = 2251 vs. *n*=2367)	8
Impact on lifestyle behaviour								
Hemphill et al. (2020)	Canada	Vancouver, Canada	Prospective cohort study (April 2017–March 2020)	109 children, age 9–16 (13 ± 2.3), 58%/42% (2019) 52%/48% (2020)	Not specified	Changes in physical activity using daily step count in young people with congenital heart disease	Decreased steps in week 13 (6417 vs 8409) and week 14 (7362 vs 10,657)	7
Pietrobelli et al. (2020)	Italy	Verona, Italy	Cohort study (10 March 2020 vs 13 May–30 July 2019)	41, age 6–18 (13 ± 3.1), 53.7%/46.3%	Quarantine (measurements after 3 weeks)	Lifestyle changes in overweight/obese young people	Decreased physical activity and a different composition of daily nutritional intake	7
Impact on vaccination rates								
Bramer (2020)	US	Michigan, US	Retrospective cohort (May 2020 vs. May 2016–2019) using electronic database	9269 (average 2016–2019) and 9539 (2020) children, age <24 months, age 2–18 years	Stay-at-home orders (starting 23 March 2020)	Changes in amount of vaccines administered	Decreased number of administered vaccines (66.6%–67.9% in 2016–2019 to 49.7% in May 2020 for children aged 5 months)	8
Chandir et al. (2020)	Pakistan	Karachi, Pakistan	Retrospective cohort (23 March–9 May 2020 vs. 23 September 2019–22 March 2020)	701,324 records, age from birth—23 months	Lockdown (starting 23 March 2020)	Vaccination coverage	Decreased number of children vaccinated during lockdown (608 832 immunisations during the baseline period and 92 492 during the lockdown)	8
McDonald et al. (2020)	UK	UK	Retrospective cohort (January—April 2020 vs. same period in 2019)	Hexavalent vaccines: 69,568 (2019) and 67,116 (2020)	Not specified	Changes in amount of vaccines administered	5.8% decrease of hexavalent, 1.0% decrease of administered MMR vaccines early 2020, 6.7% and 19.8% decrease during the pandemic	8
				MMR vaccines: 65,341 (2019) and 61,832 (2020)				
Santoli et al. (2020)	US	US	Retrospective cohort (6 January–19 April 2020 vs 7 January–21 April 2019)	Children, age <24 months and 2–18 years	Not specified	Vaccination coverage	Decreased number of ordered and administered vaccines	8
Impact on health care use								
Angoulvant et al. (2020)	France	France	Cohort study (1 January 2017–19 April 2020)	871,543 paediatric emergency department visits in 6 centres	Lockdown (18 March–19 April 19^th^ 2020)	Hospital admissions and visits before/during/after lockdown for diseases disseminated through contact (gastroenteritis, common cold, bronchiolitis, acute otitis media)	Decreased presentation of patients with several diseases during the pandemic	8
Bram et al. (2020)	US	Philadelphia US	Retrospective cohort (15 March–15 April 2020 vs same period in 2018 and 2019)	1745 individual paediatric fracture cases, age from birth—18, 57.6%/42.4% (2018–2019) 52%/48% (2020)	Home confinement (starting 16 March 2020)	Fracture incidence	Decreased fracture incidence during the pandemic (22.5 ± 9.1/day vs 9.6 ± 5.1/day)	9
Bressan et al. (2020)	Italy	Padova, Italy	Retrospective cohort (8 March–20 April 2020 vs same period in 2019)	796 (2020) and 2917 (2019) paediatric emergency department patients, age >1	Lockdown	Presentations and hospitalisations for domestic accidents	Increased visits for domestic injuries during lockdown	8
Li et al. (2020)	China	Hangzhou, China	Retrospective cohort (1 January–31 March 2020 vs same period in 2019) using electronic health records	Children, age <14	Home quarantine	Visiting rates per disease	Increased presentation for domestic injuries	8

^*^For all included papers, COVID-19 was the ‘intervention’, studies specifying disease-containment measures are mentioned here.

### Impact on mental health

#### Anxiety and depressive symptoms

The prevalence of mental health issues across studies ranged from 7.4 to 37.4% for anxiety (Chen et al., 2020; Xie et al., 2020; Zhou et al., 2020), from 6.3 to 43.7% for depressive symptoms (Zheng et al., 2020; Zhou et al., 2020), and from 6.6% (Chen et al., 2020) to 31.3% (Zhou et al., 2020) for both. One study reported that rates of anxiety were higher but that rates of depression were lower during the pandemic than before (Zheng et al., 2020).

Various risk factors for anxiety and/or depression during the pandemic were reported (Chen et al., 2020; Pınar Senkalfa et al., 2020; Saddik et al., 2020; Xie et al., 2020; Zheng et al., 2020; Zhou et al., 2020). These included increased older age, low physical activity, conflict with parents during the pandemic, lower parental education, divorced/separated parents, parents as teachers, parents with severe anxiety, (Xie et al., 2020; Zheng et al., 2020); and less optimism (Xie et al., 2020), an unbalanced learning/rest ratio, concerns about being infected with COVID-19, being alone at home on work days, limited knowledge about disease-containment strategies and COVID-19 trends and living in rural areas. Two studies reported that female sex was a risk factor for anxiety and/or depression (Chen et al., 2020; Zhou et al., 2020), one reported higher risk for males (Zheng et al., 2020) and two studies found no difference between the sexes (Pınar Senkalfa et al., 2020; Xie et al., 2020).

#### Vulnerable groups

Specific groups of children were examined in three studies (Bobo et al., 2020; Colizzi et al., 2020; Pınar Senkalfa et al., 2020). The first assessed children with cystic fibrosis (CF), and found that they experienced lower levels of anxiety than children without CF (Pınar Senkalfa et al., 2020). Among children with autism spectrum disorder (ASD), more frequent and more intense behaviour problems were reported (Colizzi et al., 2020). Among children with attention deficit hyperactivity disorder (ADHD), both worsened and improved wellbeing were reported (Bobo et al., 2020). Additionally, for some children with ADHD, home-schooling resulted in struggles to complete tasks, however, having less school-related strain also improved anxiety and self-esteem due to decreased exposure to negative feedback (Bobo et al., 2020).

### Impact on domestic violence and child abuse

#### Domestic violence

A study of police reports in Chicago, United States, showed that the proportion of child victims of domestic abuse decreased in March 2020 (*n* = 53; 2.35%) compared with 2019 (*n* = 84; 3.55%) (McLay, 2020) During enforcement of the shelter-in-place order, cases with child victims were 67% less likely.

#### Online child sexual abuse

The European Union Agency for Law Enforcement Cooperation (Europol) reported that there were increased amounts of child sexual abuse material available online during the pandemic (EUROPOL, 2020). In European countries, sharing and re-sharing of content had risen with 106% during lockdown (EUROPOL, 2020). A global network of hotlines for kids (INSAFE) reported the highest number of young people seeking contact in the last 4 years. Although exact reasons for contact were not mentioned, they were included child sexual abuse (EUROPOL, 2020).

### Impact on lifestyle behaviour

Hemphill et al. (2020) showed that physical activity levels of children with congenital heart disease decreased during the pandemic. In a study among children who were overweight or obese in Italy, children were significantly less active, with increases in screen time and sleep duration (Pietrobelli et al., 2020). The intake of snacks, red meat and sugary drinks also increased, as well as the number of meals a day (Pietrobelli et al., 2020).

### Impact on vaccination rates

Studies reported that vaccine coverage was lower during the pandemic compared to similar periods in previous years (Bramer, 2020; Chandir et al., 2020; McDonald et al., 2020; Santoli et al., 2020). In the United Kingdom, vaccination rates were already dropping in early 2020 compared with 2019, but declined further after social distancing measures were implemented (McLay, 2020). Reductions in vaccination counts in the United States were less prominent in children aged ≤24 months compared with children aged ≤18 years for non-influenza vaccines (Bramer, 2020) and for measles-containing vaccines (Santoli et al., 2020). After lockdown restrictions were lifted, vaccination coverage started to rise again (Chandir et al., 2020; McDonald et al., 2020; Santoli et al., 2020).

### Impact on health care use

Studies reported fewer paediatric emergency department (PED) visits during the pandemic than before (Angoulvant et al., 2020; Bram et al., 2020; Li et al., 2020), with reported declines up to 68% in France (Angoulvant et al., 2020) and 75% in China (Li et al., 2020).

One study showed that children aged ≥12 years were less likely to present with fractures, whilst children aged <12 were more likely to present (Bram et al., 2020). Angoulvant et al. (2020) reported that visits for diseases spread through contact were 70% lower than the expected values. Three studies, of which one during lockdown (Bressan et al., 2020), reported increased presentation for domestic injuries (Bram et al., 2020; Bressan et al., 2020; Li et al., 2020). Other reasons for more frequent PED visits were related to adolescent development and skin problems (Li et al., 2020), as well as bicycle injuries (Bram et al., 2020) and high-energy falls (Bram et al., 2020).

## Discussion

The review identified the indirect health impacts of the COVID-19 pandemic on children and adolescents. Included studies reveal that young people are experiencing new and exacerbated health challenges during the pandemic, particularly in terms of substantial mental health issues, along with increased reports of online sexual abuse and reduced physical activity among vulnerable groups. Yet there are also indications that young people may be having less contact with health care services with fewer vaccination and emergency department visits along with decreased reporting of paediatric domestic violence. Despite these indications, there is a critical lack of empirical evidence of the risk and protective factors for health impacts associated with the pandemic, as well as insufficient insight into the consequences for young people, both now and for their future health and wellbeing.

### Mental health

The most evaluated impact of the COVID-19 pandemic was on young people’s mental health. However, studies were limited by cross-sectional study design, infrequent and inconsistent use of validated measures, and the reporting of various outcomes meant that comparison across studies was constrained. Despite these limitations, it is evident that young people are vulnerable to the onset of mental health conditions, and this may be exacerbated or accelerated due to COVID-19 (Fegert et al., 2020).

While research is scarce, some negative mental health impacts of COVID-19 in children were related to information on precautionary measures and disease control during early stages of the crisis (Zhou et al., 2020). Only one study compared quarantined and not-quarantined children, showing higher rates of psychological distress in the first group (Saurabh and Ranjan, 2020). Likewise, only one study compared mental health outcomes to pre-pandemic norms in the population (Zheng et al., 2020). In the present review, one study reported that males were more likely to experience anxiety (Zheng et al., 2020), which contradicts previous evidence on sex differences in mental health among children and adolescents, demonstrating higher anxiety rates in females (Bender et al., 2012). These findings are comparable to studies on the psychological impact of previous emergencies and disasters (e.g. epidemics, terrorist attacks, hurricanes, floods) (Danese et al., 2020; Fan et al., 2020; Sprang and Silman, 2013). Young people may mostly experience transient symptoms such as fear, detachment, numbness and feelings of worry that an event will reoccur (Danese et al., 2020). Nevertheless, a smaller proportion will eventually develop anxiety and/or depression, substance use or post-traumatic stress disorder (Danese et al., 2020; Sprang and Silman, 2013). Taken together with the present review, there are indications that children and adolescents experienced substantial symptoms of anxiety and depression during this pandemic. Furthermore, for children with existing co-morbidities and/or neurodiversity, such as ASD or ADHD there are added complexities, which may relate to disruptions to routine and/or school closures. It is vital that the implications of measures to reduce the spread of the pandemic are considered for children and young with neurodiversity.

### Domestic violence and child abuse

There was extremely limited insight into how young people’s exposure to domestic violence and abuse has been impacted by COVID-19. The study on domestic violence only contained reports from a single police district, and did not elaborate on types of violence and abuse experienced by child victims. Likewise, the Europol report did not provide exact victim or perpetrator characteristics. Furthermore, while police reported cases purportedly decreased, there are known issues around decreased reporting during the COVID-19 pandemic (Đapić et al., 2020). As children spend the majority of week-days at school, teachers have a significant role in the detection of abuse (Đapić et al., 2020). School closures may therefore contribute to declines in reported cases of child abuse (Đapić et al., 2020). Further, social distancing in the community more broadly may also be limiting the observations of others who are usually present in a child’s everyday environment (Đapić et al., 2020).

Decreased reported abuse to authorities does not align with the increase of anecdotal evidence, media reports and phone calls for domestic violence to different helplines around the world (Đapić et al., 2020; EUROPOL, 2020; Peterman et al., 2020). This trend has been present during previous crises. For instance, during the Ebola epidemic, girls in West Africa were exploited for sex (Đapić et al., 2020; Elston et al., 2017). Although evidence of the impact of COVID-19 on violence and abuse experienced by young people is limited, a lack of evidence cannot be construed as proof of the contrary, and the potential detrimental impact has been demonstrated in research on previous crises (Đapić et al., 2020). Indeed, the impacts of abuse, both physical and sexual, are intertwined with other health impacts, including adult anxiety, depression and self-harm, post-traumatic stress disorder and substance use (Fergusson et al., 2008). Thereby, surveillance and detection of child abuse is needed to prevent long-term health impact and address the life-long impacts of traumatic experiences.

### Lifestyle behaviour

There is limited understanding of how the social isolation measures have impacted physical activity and nutrition of children and adolescents (Margaritis et al., 2020), and the studies included in this review were limited to specific populations. Research on healthy and obese children, comparing school days with holiday periods, has shown weight gain during holiday periods, for reasons unknown (Franckle et al., 2014; Von Hippel et al., 2007; Wang et al., 2015). Furthermore, it is not clear, if and to what extent isolation measures may interact with other social determinants that are known to be indicators of physical activity and nutrition (Margaritis et al., 2020). It is quite likely that this will disproportionately impact young people growing up in low-income households and poverty, and food insecurity is expected to be exacerbated due to school closure, for those whom received a significant proportion of their healthy daily nutrition at school (Dunn et al., 2020; Kinsey et al., 2020; Van Lancker and Parolin, 2020). Moreover, food insecurity is associated with short-term fatigue and decreased immunity (Dunn et al., 2020), as well as worse academic performance, and poorer physical and mental health in the long-term (Dunn et al., 2020; Van Lancker and Parolin, 2020). There is also an increased risk of obesity (Kinsey et al., 2020). The impact of decreased food access due to the pandemic on paediatric mortality has been modelled for LMICs. This study showed that increased wasting of 10% and 50% over 6 months would lead to 253,500 and 1,157,000 additional child deaths, which would amount to 18–23% increased mortality (Roberton et al., 2020). Further research is needed to assess to what extent (prolonged) school closure has severe consequences, which could then determine if these outweigh the risks of reopening.

### Vaccination rates and health care use

This review shows that vaccination rates and the use of paediatric health care materially decreased during the pandemic. Research on prior health care crises showed similar findings, such as reductions in vaccine coverage during the Ebola outbreak in 2014 (Elston et al., 2017) and lower PED visit rates during the SARS epidemic in 2003 (Chu et al., 2008). To improve the understanding of the impacts on health care, the full spectrum of paediatric health care should be assessed. For example, reorganisation of patient care in hospitals, away from the emergency department, could have impacted visits rates to the emergency department. Likewise, parents might have preferred to take their children to local clinics instead of PED for minor injuries due to fear of exposure to COVID-19 in the emergency department. Furthermore, fracture cases could have been treated by adult orthopaedic surgeons, because paediatric orthopaedic surgeons were not available at the nearest health care facility.

### Implications for future health

The findings from this review reinforce concerns raised by experts young people will face substantial health impacts for their current and future health as a result of the pandemic (Green, 2020; Van Lancker and Parolin, 2020). While the causal nature indirect health impacts of the COVID-19 pandemic could not be established in this review, the physical and psychosocial health impacts identified may have serious ongoing ramifications for the healthy development of children and adolescents. During adolescence, for instance, quality of life is affected by (mental) health problems (Hendekçi and Bilgin, 2020; Zhou et al., 2020); however, mental health issues frequently persist into adulthood particularly if not identified and sufficiently addressed (Danese et al., 2009). This emphasises the importance of identifying and responding to the reported increases in mental health concerns among young people, as it often takes individuals with early-onset problems a decade or more, to seek initial treatment (Kessler et al., 2007). Such delays in access to treatment can have several long-term implications, increased smoking and substance use, eating disorders, obesity, social and educational maladjustments, severe depression with self-harm (Kessler et al., 2007; Petito et al., 2020). It is critical that these long-term consequences are prevented through effective early detection and intervention (Kessler et al., 2007; Petito et al., 2020) Likewise, violence and abuse of children are risk factors for poor current and future physical and mental health outcomes (Norman et al., 2012), and declines in health care use during childhood may have adverse long-term consequences, as it is a critical developmental period for establishing health behaviours and access to care (Patton et al., 2016, 2018). Moreover, declines in vaccination coverage can have significant consequences for herd immunity, which could result in outbreaks of preventable infectious diseases (Bramer, 2020; Santoli et al., 2020).

### Strengths and limitations

This review reports on the best available evidence on the early impact of the COVID-19 pandemic on children and adolescents. A broad cross-section of outcomes was identified and there was geographic heterogeneity between studies, allowing broader applicability of the results.

This study has several limitations. First, differences between studies in design, setting, definitions, population and outcome restricted the comparability between studies and prohibited a meta-analysis. Second, the review only contained observational studies, several of which were cross-sectional. As such, we could not assess whether the reported relationships were causal. Third, the scope of this review was limited to initial evidence of impacts of COVID-19. Fourth, although children and adolescents are two different groups with wide age ranges, the data presented by the included articles often did not include disaggregation by age. Lastly, as the COVID-19 pandemic has had a worldwide impact, the gathered information might be incomplete due to possibly excluded relevant papers, written in languages other than English.

### Implications for policy and practice

This review contributes to our understanding of how child and adolescent health is indirectly affected by COVID-19. Identified impacts provide a range of short-term consequences and potential long-term repercussions, that should be considered as a starting point for developing targeted interventions. Evidence suggests that while not every child is at risk of exposure to deleterious outcomes, those who are already vulnerable pose higher risk, which could amplify existing inequalities. Therefore, a key policy priority should be to identify those at-risk, including young people with pre-existing mental health conditions, history of domestic and/or sexual abuse. Protective measures should be implemented to prevent deterioration of their health, while simultaneously maintaining a healthy status for non-vulnerable children. Furthermore, health changes (e.g. new diagnoses, vaccination status, dietary and physical activity status, abuse reports) should be prospectively monitored, enabling pre- and post-pandemic comparison. With this, assistance could be better targeted and negative health outcomes of identified impacts can be mitigated. Indeed, at a policy level, there is a need for resources that will ensure negative impacts are monitored and addressed. Subsequently, policy makers should seek to determine that repercussions do not outweigh potential benefits of disease-containment measures.

In conclusion, this review demonstrates that children and adolescents are facing several indirect health consequences of the COVID-19 pandemic, including mental health issues, reduced access to health care, poorer lifestyle habits and increases in online sexual abuse. These findings provide impetus for policy makers and health care organisations in developing effective interventions to protect young people during crises and to ensure their future health and wellbeing.

## Supplemental Material

sj-pdf-1-chc-10.1177_13674935211059980 – Supplemental Material for The indirect health impacts of the COVID-19 pandemic on children and adolescents: A review Click here for additional data file. Supplemental Material, sj-pdf-1-chc-10.1177_13674935211059980 for The indirect health impacts of the COVID-19 pandemic on children and adolescents: A review by Tina GA Oostrom, Patricia Cullen and Sanne AE Peters in Journal of Child Health Care

sj-pdf-2-chc-10.1177_13674935211059980 – Supplemental Material for The indirect health impacts of the COVID-19 pandemic on children and adolescents: A review Click here for additional data file. Supplemental Material, sj-pdf-2-chc-10.1177_13674935211059980 for The indirect health impacts of the COVID-19 pandemic on children and adolescents: A review by Tina GA Oostrom, Patricia Cullen and Sanne AE Peters in Journal of Child Health Care

sj-pdf-3-chc-10.1177_13674935211059980 – Supplemental Material for The indirect health impacts of the COVID-19 pandemic on children and adolescents: A review Click here for additional data file. Supplemental Material, sj-pdf-3-chc-10.1177_13674935211059980 for The indirect health impacts of the COVID-19 pandemic on children and adolescents: A review by Tina GA Oostrom, Patricia Cullen and Sanne AE Peters in Journal of Child Health Care
