# Supplementary material for: The indirect health impacts of the COVID-19 pandemic on children and adolescents: A review
Source: J Child Health Care. 2022 Mar 10;27(3):488–508. doi: 10.1177/13674935211059980 (PMC8919137; doi:10.1177/13674935211059980)
Supplement: sj-pdf-1-chc-10.1177_13674935211059980 – Supplemental Material for The indirect health impacts of the COVID-19 pandemic on children and adolescents: A review [file sj-pdf-1-chc-10.1177_13674935211059980.pdf]

**Table I Search and selection strategy for a narrative review of indirect impacts of the COVID-19 pandemic on children and adolescents**		
Search keywords	*Domain:* child*, kid*, adolescen*, infan*, minor*, teen*, juvenile*, youth, youths, youngster*, young people*, pediatri*, paediatr*, toddler*, kindergart*, preschool*, playgroup*, play-group*, playschool*, schoolchild*, prepube*, preadolescen*, puberty*, pube* school, junior high*, high school*, senior high*, boy, boys, boyhood, girl, girls, girlhood<br>*Determinant:* COVID-19*, COVID19*, COVID 19*, coronavirus*, corona virus*, SARS2, SARS-Cov-2, Sars Cov 2, SARS corona virus 2, SARS coronavirus 2, Cov 2, Cov2, nCov, 2019nCov, nCov 2019, severe acute respiratory syndrome cov 2, severe acute respiratory syndrome cov2	
Other sources	• World Health Organization (WHO)<br>• Pan American Health Organization (PAHO)<br>• Centers for Disease Control and Prevention (CDC)<br>• European Centre for Disease Prevention and Control (ECDC)<br>• Chinese Center for Disease Control and Prevention (CCDC)	• National Institutes of Health (NIH)<br>• National Health Service (NHS)<br>• National Institute for Public Health and the Environment (RIVM)<br>• MedRxiv<br>• BioRxiv<br>• Europol
Inclusion criteria	• Articles focused on children and/or adolescents	• Articles discussing one or multiple ways in which the COVID-19 pandemic has (had) an impact
Exclusion criteria	• Articles written before 2019<br>• Articles focused on the medical aspects of COVID-19<br>• Articles not focused on children and/or adolescents	• Articles not presenting evidence-based material<br>• Articles not written in English<br>• Full-text unavailability
