# Supplementary material for: The indirect health impacts of the COVID-19 pandemic on children and adolescents: A review
Source: J Child Health Care. 2022 Mar 10;27(3):488–508. doi: 10.1177/13674935211059980 (PMC8919137; doi:10.1177/13674935211059980)
Supplement: sj-pdf-2-chc-10.1177_13674935211059980 – Supplemental Material for The indirect health impacts of the COVID-19 pandemic on children and adolescents: A review [file sj-pdf-2-chc-10.1177_13674935211059980.pdf]

Table II Search string for databases	
Pubmed/MEDLINE	(((((((((((((((((((((((((((((((((((Child*[Title/Abstract]) OR (Kid[Title/Abstract])) OR (Kids[Title/Abstract])) OR (Adolescen*[Title/Abstract])) OR (Infan*[Title/Abstract])) OR (Minor*[Title/Abstract])) OR (Teen*[Title/Abstract])) OR (Juvenil*[Title/Abstract])) OR (Youth[Title/Abstract])) OR (Youths[Title/Abstract])) OR (Youngster*[Title/Abstract])) OR (Young people*[Title/Abstract]))) OR (Pediatri*[Title/Abstract])) OR (Paediatr*[Title/Abstract])) OR (Toddler*[Title/Abstract])) OR (Kindergart*[Title/Abstract])) OR (Preschool*[Title/Abstract])) OR (Playgroup*[Title/Abstract])) OR (Play-group*[Title/Abstract])) OR (Playschool*[Title/Abstract])) OR (Schoolchild*[Title/Abstract])) OR (Toddler*[Title/Abstract])) OR (Kindergart*[Title/Abstract])) OR (Prepube*[Title/Abstract])) OR (Preadolescen*[Title/Abstract])) OR (Puberty[Title/Abstract])) OR (Pube* School[Title/Abstract])) OR (Junior high*[Title/Abstract])) OR (High School*[Title/Abstract])) OR (Senior high*[Title/Abstract])) OR (boy[Title/Abstract])) OR (boys[Title/Abstract])) OR (boyhood[Title/Abstract])) OR (girl[Title/Abstract])) OR (girls[Title/Abstract])) OR (girlhood[Title/Abstract]) OR (Adolescent[MeSH Terms])) OR (Child[MeSH Terms])) OR (infant[MeSH Terms])) AND ((((((((((((((((((((COVID-19*[Title/Abstract]) OR (COVID 19*[Title/Abstract])) OR (COVID19*[Title/Abstract])) OR (Coronavirus*[Title/Abstract])) OR (Corona virus*[Title/Abstract])) OR (SARS-CoV-2[Title/Abstract])) OR (Sars Cov 2[Title/Abstract])) OR (SARS corona virus 2[Title/Abstract])) OR (SARS coronavirus 2[Title/Abstract])) OR (Severe acute respiratory syndrome cov 2[Title/Abstract])) OR (Severe acute respiratory syndrome cov2[Title/Abstract])) OR (nCov[Title/Abstract])) OR (2019nCov[Title/Abstract]))) OR (nCov 2019[Title/Abstract]) OR (Cov 2[Title/Abstract])) OR (Cov2[Title/Abstract])) OR (SARS2[Title/Abstract]))) OR (Severe acute respiratory syndrome cov 2[Title/Abstract])) OR (Severe acute respiratory syndrome cov2[Title/Abstract])) OR (COVID-19[Supplementary Concept])) OR (severe acute respiratory syndrome coronavirus 2[Supplementary Concept])) OR (Coronavirus[Mesh:NoExp] OR Coronavirus Infections[Mesh:NoExp])) AND ("2019/12"[Date - Entrez] : "3000"[Date - Entrez])
Embase	(child*:ti,ab,kw OR adolescen*:ti,ab,kw OR infan*:ti,ab,kw OR juvenil*:ti,ab,kw OR 'young people':ti,ab,kw OR toddler:ti,ab,kw OR kindergart*:ti,ab,kw OR 'preschool*':ti,ab,kw OR playground*:ti,ab,kw OR 'school child':ti,ab,kw OR prepube*:ti,ab,kw OR preadolescen*:ti,ab,kw OR puberty:ti,ab,kw OR 'high school*':ti,ab,kw OR boy:ti,ab,kw OR girl:ti,ab,kw OR 'juvenile'/exp OR boys:ti,ab,kw OR boyhood:ti,ab,kw OR girls:ti,ab,kw OR girlhood:ti,ab,kw OR minor*:ti,ab,kw OR teen*:ti,ab,kw OR youth:ti,ab,kw OR youths:ti,ab,kw OR youngster*:ti,ab,kw OR 'senior high*':ti,ab,kw OR 'junior high*':ti,ab,kw OR 'play-group*':ti,ab,kw OR 'playschool*':ti,ab,kw OR playschool*:ti,ab,kw OR pediatri*:ti,ab,kw OR paediatr*:ti,ab,kw OR 'pube* school':ti,ab,kw) AND (('coronavirinae'/exp OR 'coronavirus infection'/de OR coronavirus*:ti,ab,kw OR 'corona virus*':ti,ab,kw OR 'pneumonia virus*':ti,ab,kw OR cov:ti,ab,kw OR ncov:ti,ab,kw) AND (outbreak:ti,ab,kw OR wuhan:ti,ab,kw) OR covid19:ti,ab,kw OR 'covid 19':ti,ab,kw OR ((coronavirus*:ti,ab,kw OR 'corona virus*':ti,ab,kw) AND 2019:ti,ab,kw) OR 'sars cov 2':ti,ab,kw OR sars2:ti,ab,kw OR 'coronavirus*':ti,ab,kw OR 'corona virus*':ti,ab,kw OR 'ncov 2019':ti,ab,kw OR ncov:ti,ab,kw OR 'sars coronavirus 2':ti,ab,kw OR 'sars corona virus 2':ti,ab,kw OR 'severe acute respiratory syndrome cov 2':ti,ab,kw OR 'severe acute respiratory syndrome cov2':ti,ab,kw) AND [2019-2020]/py
