# Supplementary material for: The indirect health impacts of the COVID-19 pandemic on children and adolescents: A review
Source: J Child Health Care. 2022 Mar 10;27(3):488–508. doi: 10.1177/13674935211059980 (PMC8919137; doi:10.1177/13674935211059980)
Supplement: sj-pdf-3-chc-10.1177_13674935211059980 – Supplemental Material for The indirect health impacts of the COVID-19 pandemic on children and adolescents: A review [file sj-pdf-3-chc-10.1177_13674935211059980.pdf]

**Table III** **Newcastle – Ottawa Quality Assessment Scale (NOS)**				
Cohort studies (NOS max. 9)				
**First author**	**Selection**	**Comparability**	**Outcome**	**NOS Score**
Angoulvant	****	*	***	8
Bram	****	**	***	9
Bramer	****	*	***	8
Bressan	****	*	***	8
Chandir	****	*	***	8
Hemphill	****	*	**	7
Li	****	*	***	8
McDonald	****	*	***	8
McLay	****	*	***	8
Pietrobelli	***	**	**	7
Santoli	****	*	***	8
Cross-sectional studies (NOS max. 10)				
Bobo	****	*	**	7
Buzzi	**	-	*	3
Chen	****	**	**	8
Colizzi	****	**	**	8
Saddik	****	**	**	8
Saurabh	***	**	*	6
Senkalfa	****	*	**	7
Xie	****	*	**	7
Zheng	*****	**	**	9
Zhou	*****	*	**	8
